# A Rare Case of Locally Acquired Malaria in Lebanon: A Public Health Concern

**DOI:** 10.7759/cureus.76298

**Published:** 2024-12-24

**Authors:** Douaa Abou Hamdan, Dalia Hassan, Jamil Mourad, AbdulKarim M El Karaaoui

**Affiliations:** 1 Infectious Diseases Department, Lebanese University, Beirut, LBN; 2 Infection Control Department, Bekaa Hospital, Bekaa, LBN; 3 Pharmacy, Biology, and Laboratory Department, Bekaa Hospital, Bekaa, LBN; 4 Clinical Pathology and Laboratory Department, Bekaa Hospital, Bekaa, LBN

**Keywords:** autochthonous malaria, locally transmitted malaria, malaria in lebanon, nosocomial malaria, plasmodium falciparum

## Abstract

Malaria, a mosquito-borne disease caused by five plasmodium species, still has a life-threatening risk worldwide. Clinical manifestations can range from mild nonspecific symptoms to severe disease. In non-endemic regions, sporadic cases frequently pose significant challenges to health workers as delayed diagnosis can lead to serious consequences and even death. In this study, we report the first documented case of locally transmitted malaria in Lebanon involving a patient with no recent travel history presenting with fever, chills, and headache. The patient was found to have anemia and thrombocytopenia, and a positive malaria antigen test and peripheral smear revealed the presence of banana-shaped gametocytes and intracellular rings confirming the diagnosis of plasmodium falciparum. She was treated successfully with intravenous artesunate, and she fully recovered.

## Introduction

Malaria, a mosquito-borne disease caused by five protozoan species within the Plasmodium genus, remains a significant global health concern. Malaria presents with a diverse range of symptoms, from mild to severe. Common manifestations include fever, headache, fatigue, and chills. In more severe cases, complications such as anemia and thrombocytopenia can arise, potentially leading to life-threatening conditions [1].

While the number of malaria cases has declined in recent decades, it still poses a life-threatening risk, particularly due to Plasmodium falciparum, which accounts for the majority of malaria-related deaths worldwide. According to the 2024 World Health Organization Malaria Report, there were an estimated 263 million malaria cases globally in 2023, resulting in approximately 597,000 deaths [1]. The Eastern Mediterranean Region has seen the most significant increase in malaria cases, with a 57% rise since 2021 [1]. Malaria transmission in non-endemic regions is uncommon, but sporadic cases occur globally. These cases often present significant challenges to local and public health authorities due to delayed diagnosis and treatment, leading to severe consequences [2].

In non-endemic areas, the majority of malaria cases are acquired through international travel. However, there are instances where patients report no recent travel history, necessitating investigations into alternative transmission mechanisms. Several potential avenues for infection exist, including the introduction of competent mosquito vectors. These vectors may be imported, as in cases of "airport malaria" or "baggage malaria," especially in regions with suitable climatic conditions. Additionally, nosocomial transmission, where infection occurs within healthcare settings, is another potential route of introduction [3].

Lebanon has remained free from local malaria transmission since 1963 [4]. In 2012, the country reported 115 imported malaria cases, with half of them caused by Plasmodium falciparum. Although a malaria case was reported in Sidon, Lebanon, in 2019 by the Ministry of Public Health, the source of the infection (whether local or imported) remains undetermined due to the lack of additional public health data [5].

In this study, we present the first documented case of locally transmitted malaria in Lebanon involving a patient with no recent travel history. Our aim is to emphasize the importance of heightened surveillance in non-endemic regions and to raise awareness among healthcare professionals about the potential resurgence of malaria.

## Case presentation

We present the case of a previously healthy 27-year-old Lebanese woman with no known food or drug allergies. The patient, a habitual hookah smoker, presented to the emergency department of our Bekaa hospital complaining of severe headache, high-grade fever (up to 39 °C), chills, nausea, diffuse bone pain, and fatigue that had developed several days prior to admission.

The patient reported no recent travel history and no contact with individuals from malaria-endemic regions or residing near airports. She experienced mild dysuria but denied any respiratory symptoms or diarrhea.

Upon further questioning, the patient revealed that two weeks prior to her symptoms, she had undergone a dilation and curettage (D&C) procedure in a clinic that lacked adequate safety precautions. She also mentioned that another patient from Africa was present at the clinic during her procedure.

Upon presentation, the patient exhibited a fever of 39 °C, normotensive blood pressure (125/70 mmHg), a rapid pulse rate of 113 beats per minute, and maintained oxygen saturation of 96% on room air. Physical examination revealed a pale and lethargic patient with intact neurological function and a soft, nontender abdomen. Cardiovascular and respiratory auscultation was unremarkable.

Initial hematological and biochemical laboratory results indicated severe anemia, thrombocytopenia, elevated C-reactive protein (CRP), and positive urine analysis, as detailed in Table 1. A cerebrospinal fluid (CSF) analysis was performed to rule out meningitis as a cause of the severe headache and fever. The CSF analysis was negative, excluding any neurological infection.

**Table 1:** Hematological and biochemical laboratory data on admission

Parameter	Value	Reference range
WBC	8500/µl	4000-11000
Neutrophils	73%	40-65
Hemoglobin	9.4 g/dl	12-16 (female)
Platelets	66000/µl	150000-450000
Creatinine	0.73 mg/dl	0.55-1.02 (female)
CRP	127.7 mg/L	<5
INR	1.46	0.8-1.1
GGT	20 UI/L	<38 (female)
Total Bilirubin	3.2 mg/dl	0.3-1.2
AST	79 U/L	<35 (female)
ALT	49 U/L	<35 (female)
Urine analysis: WBCs	Numerous/HPF	0-5
Urine analysis: RBCs	10-15/HPF	0-5
CSF: WBCs	10/µl	0-5
CSF: RBCs	10/µl	0-5
CSF: proteins	0.45 g/l	0.15-0.45
CSF: glucose	62 mg/dl	
CSF culture	No growth after 48 hours	No growth

**Figure 1:**
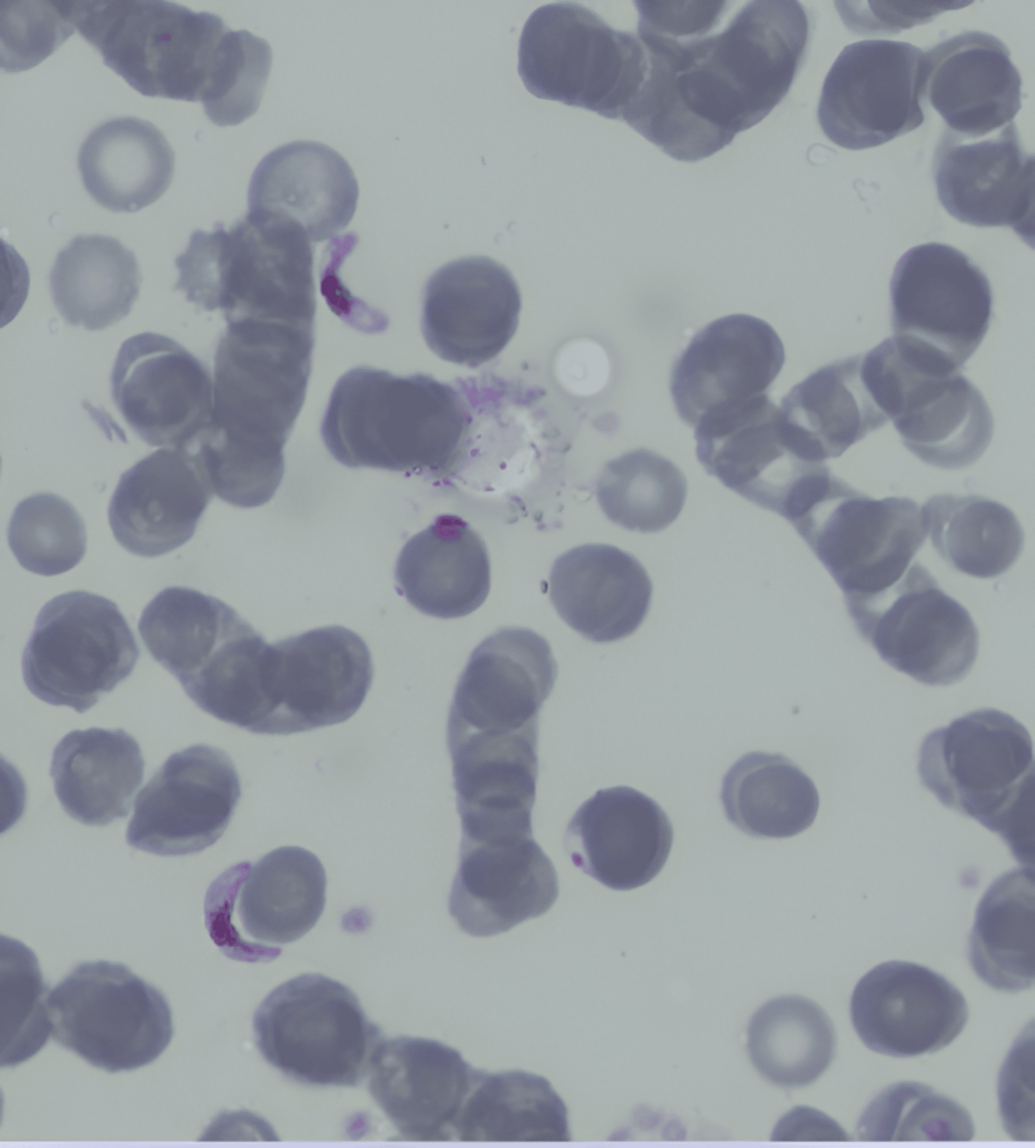
Peripheral blood smear showing two banana-shaped gametocytes

**Figure 2:**
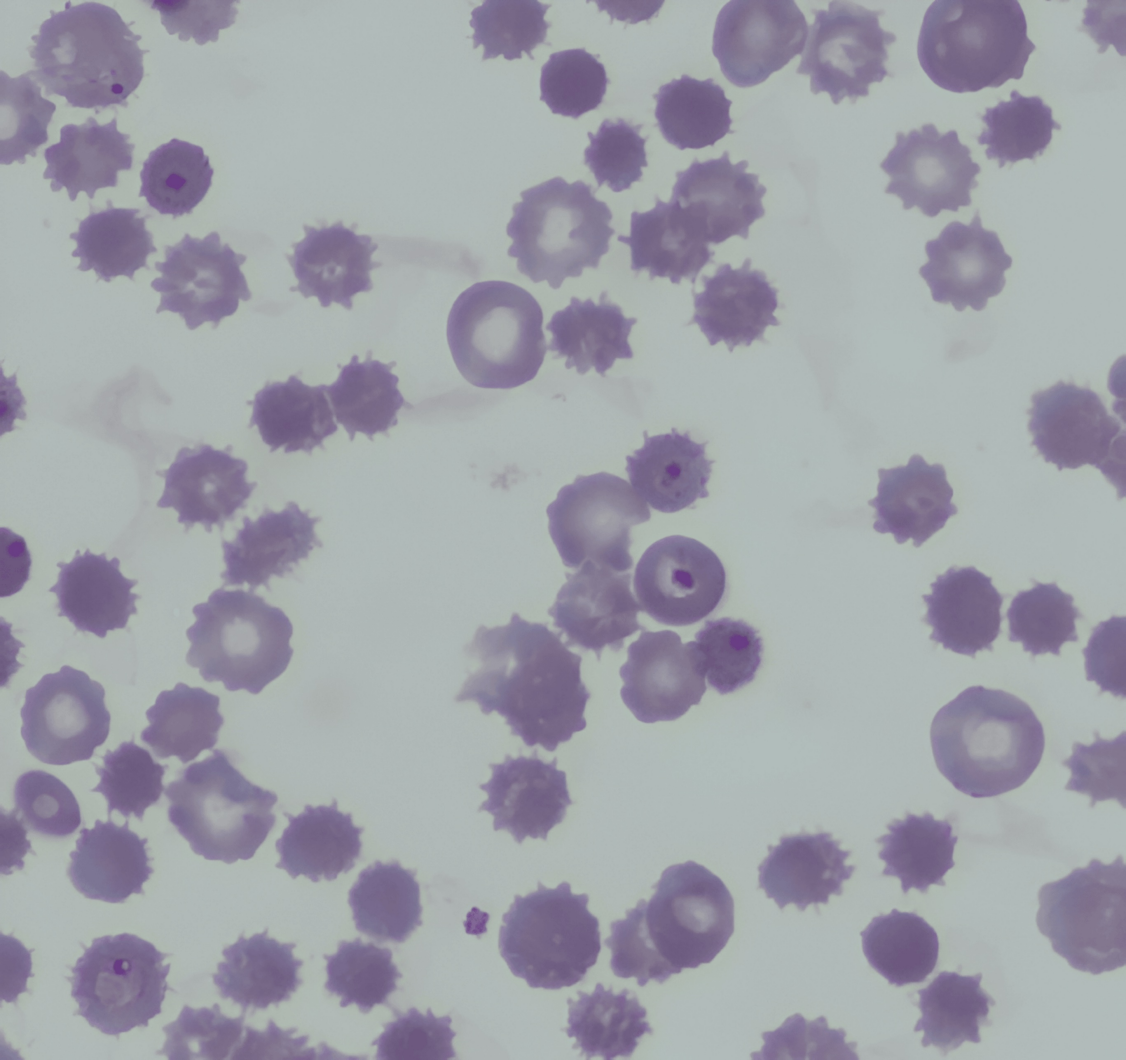
Peripheral blood smear showing intracellular rings

Empirical antibiotic therapy with ceftriaxone was initiated, along with blood transfusion and hydration. Following the identification of multidrug-resistant Klebsiella pneumoniae in the urine culture, the antibiotic regimen was escalated to meropenem. Despite six days of treatment, the patient's fever, anemia, and thrombocytopenia persisted. Subsequent laboratory investigations, including peripheral blood smear, rapid malaria antigen test, and parasitemia, yielded unexpected results, confirming a diagnosis of malaria, as detailed in Table 2, Figure 1, and Figure 2.

**Table 2:** Blood film inspection, malaria rapid antigen test, and parasitemia of the patient on day 6 of admission

Erythrocytes	Microcytosis, intracellular rings, and banana-shaped gametocytes present; Numerous target cells present; No schistocytes or spherocytosis present
Leukocytes	Normal differential, size, and shape; No atypical cells (neutrophils 65%, lymphocytes 19%)
Thrombocytes	Few platelet aggregates seen
Conclusion and interpretation	The presence of rings and gametocytes is diagnostic of Plasmodium falciparum.
Malaria antigen rapid test	Positive
Percentage parasitemia (Plasmodium)	3%

Intravenous Artesunate therapy was initiated with a loading dose of 120 mg at 0, 12, and 24 hours followed by 120 mg daily doses for three days. Subsequently, oral artemether-lumefantrine (Coartem) at a dose of 80 mg/480 mg two times a day was administered.

By day six of treatment, the fever subsided, the parasitemia decreased to 0.9%, hemoglobin stabilized at 8.7 mg/dl, and platelet count normalized to 160,000, The patient was discharged after completing a seven-day course of therapy, with a follow-up complete blood count and parasitemia tests scheduled as outpatient care.

Seven days post-treatment, the patient was asymptomatic and completely recovered. Laboratory results revealed a hemoglobin level of 7.8 mg/dl, likely due to delayed hemolysis associated with Artesunate. The blood smear was clear of parasites, but the rapid malaria antigen test remained positive.

## Discussion

Autochthonous malaria refers to locally acquired malaria, either directly from an imported case or through a locally bred mosquito infected by a traveler. Odyssean malaria (baggage malaria and airport malaria), a specific type of autochthonous malaria, occurs when a traveler brings an infected mosquito, which then bites a local resident. Less common types of autochthonous malaria include induced malaria, acquired through organ transplants or blood transfusions, and nosocomial malaria, contracted in healthcare settings. Cryptic malaria is another type where the source of infection remains unidentified. These less conventional forms of malaria can be more severe due to their unexpected nature [6].

This case report presents the first instance of locally acquired malaria in a patient with no history of travel to endemic regions, no exposure to travelers from endemic areas, and no history of blood transfusions or organ transplants. This suggests that the infection was likely acquired through contact with infected blood during a D&C procedure performed in a non-sterile clinic setting. The patient reported that an African patient, who was likely infected, had been treated in the clinic prior to her procedure. Unfortunately, testing and surveillance of the African patient were not possible. Alternatively, the infection may have been transmitted by an Anopheles mosquito, which can thrive in warm weather conditions, especially during the summer months in Lebanon.

A diagnosis of malaria typically involves microscopic examination of blood smears to identify malarial parasites. Rapid diagnostic tests (RDTs) can be used if microscopy is unavailable while PCR testing provides a definitive diagnosis and species identification. Timely diagnosis depends on a high index of suspicion from the treating physician. Patients with recent travel history to endemic regions are often considered at risk, but cases with no apparent exposure can delay diagnosis. Raising awareness among healthcare providers about the possibility of locally acquired malaria is crucial for timely diagnosis and appropriate treatment [7].

The treatment of malaria depends on the severity of the infection and the specific parasite involved. For uncomplicated, mild malaria caused by Plasmodium falciparum, artemisinin-based combination therapy (ACT) is the first-line treatment. Alternative options include atovaquone-proguanil or quinine sulfate with doxycycline. For chloroquine-sensitive parasites like Plasmodium vivax and Plasmodium oval, hydroxychloroquine remains effective [8,9]. Severe malaria, often caused by Plasmodium falciparum, requires immediate intravenous artesunate treatment [10]. Our patient received intravenous Artesunate for the first three days and then was switched when stable to oral Artemether-Lumefantrine. The patient responded well to treatment, with normalized laboratory tests and resolution of fever. Follow-up as an outpatient confirmed sustained clinical improvement.

The emergence of cholera in Syria and dengue and malaria in Pakistan underscores how crises can create conditions conducive to the spread of deadly diseases. These outbreaks pose significant public health risks, especially in countries with weakened healthcare systems and limited resources. Lebanon, too, has faced years of economic hardship, conflict, and environmental degradation, leaving it vulnerable to infectious disease outbreaks [11].

While autochthonous malaria cases have been reported in various countries, including the US, Germany, and Spain [2,6,12], the case in Lebanon is of particular concern due to the potential for widespread transmission and the country's limited resources for surveillance and control.

This study aims to raise awareness among healthcare professionals about the importance of maintaining a high index of suspicion for malaria, even in patients with no recent travel history. Given the increasing global travel and migration trends, including travel to endemic regions, it is crucial for healthcare providers to consider malaria in their differential diagnosis. Additionally, strict adherence to sterilization protocols during procedures involving blood exposure is essential to prevent nosocomial transmission of infectious diseases.

## Conclusions

Malaria, a potentially fatal disease, is caused by parasites transmitted to humans through the bite of infected female Anopheles mosquitoes. The clinical presentation of malaria can vary depending on the parasite species, the region, and the individual’s immunity. Microscopic examination of blood smears remains the gold standard for diagnosing malaria. Healthcare providers in regions traditionally considered malaria-free should maintain a high index of suspicion for malaria in febrile and anemic patients, even in the absence of a travel history. Public health officials should investigate the presence of Anopheles mosquitoes in areas where local malaria cases are confirmed. Additionally, the Ministry of Public Health should emphasize and enforce stringent sterilization standards for procedures performed outside of hospital settings, particularly in gynecological clinics.
